# Unexpected Lung Collapse Following Chest Tube Insertion for Pneumothorax Drainage in an Intubated Patient Due to Dislocation of the Endotracheal Tube

**DOI:** 10.7759/cureus.55236

**Published:** 2024-02-29

**Authors:** Christos Voucharas, Georgios Tagarakis, Angeliki Vouchara

**Affiliations:** 1 Department of Cardiothoracic Surgery, AHEPA University Hospital, Aristotle University of Thessaloniki, Thessaloniki, GRC; 2 Department of Surgery, AHEPA University Hospital, Aristotle University of Thessaloniki, Thessaloniki, GRC

**Keywords:** barotrauma, heart surgery, pneumothorax, atelectasis, postoperative lung collapse, inadvertent endotracheal intubation

## Abstract

A 67-year-old male patient was admitted to the intensive care unit following an uncomplicated heart operation. The initial postoperative chest X-ray revealed a total pneumothorax on the left side. Despite drainage of the left pleural space, a subsequent chest X-ray unexpectedly showed opacification of the left hemithorax. Partial withdrawal of the endotracheal tube resulted in complete expansion of the left lung. It is important to always consider the possibility of endotracheal tube dislocation in all intubated patients.

## Introduction

Tube displacement is a relatively common complication in patients undergoing tracheal intubation [1,2]. Misplacement of the tracheal tube into the right main bronchus may occur due to deep insertion by the anesthesiologist or as a result of head and neck movements during patient mobilization. The situation can be easily managed if the anesthesia provider properly secures the endotracheal tube to prevent displacement during patient transfer or radiogram examination, carefully reviews the radiogram for correct tube placement, and auscultates bilateral breath sounds [3].

Misplacement of the tube can lead to atelectasis (obstructive collapse) of the left lung and/or pneumothorax of the right lung due to overinflation. These complications can result in a decrease in blood pressure and oxygen saturation levels [1,4].

## Case presentation

**Figure 1:**
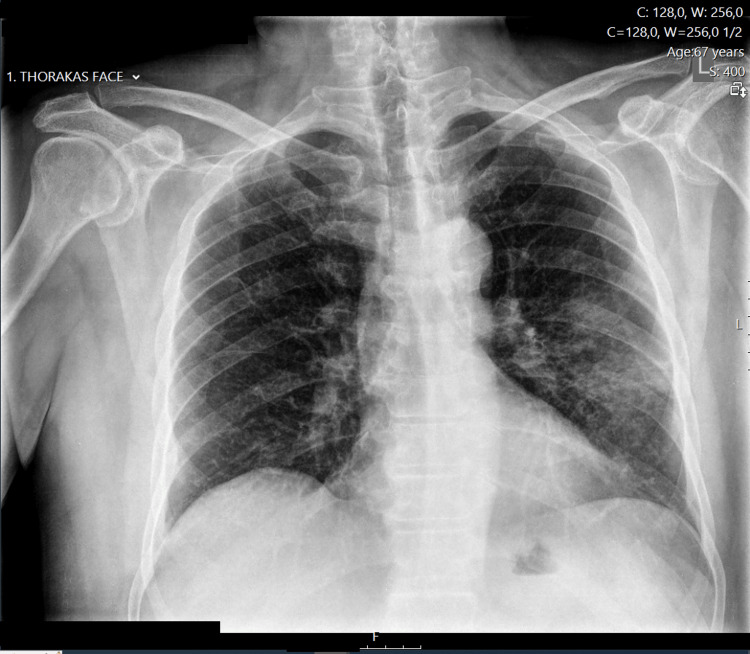
Preoperative chest X-ray.

**Figure 2:**
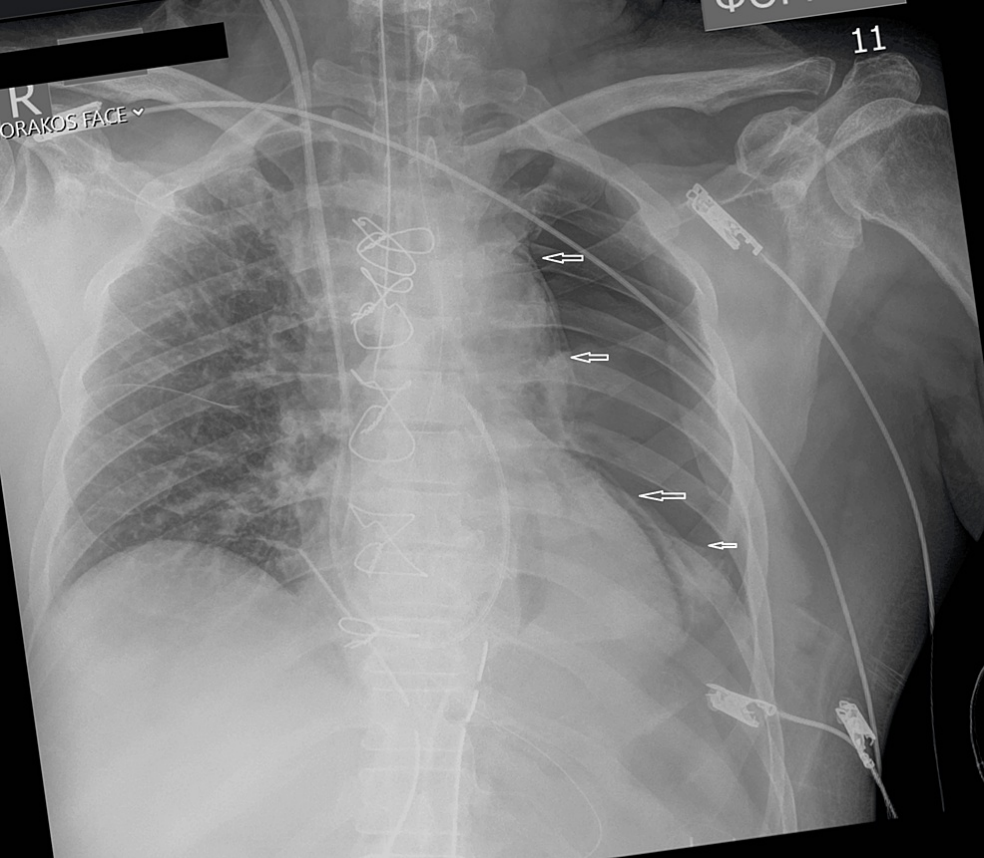
Initial portable postoperative chest radiograph showing total left pneumothorax. The arrows indicate the line of pneumothorax.

A 67-year-old patient underwent mitral valve replacement and single coronary bypass surgery under general anesthesia and the use of cardiopulmonary bypass. The pleura was intact on both sides during surgery. The procedure was uncomplicated, and the patient was transferred to the intensive care unit. The first postoperative chest X-ray revealed a complete pneumothorax on the left side. The pneumothorax was attributed to barotrauma or inadvertent puncture of the left-side pleura during chest wire application. Figure 1 shows the preoperative radiograph, while Figure 2 depicts the initial postoperative chest X-ray with a collapsed left lung. The patient's respiratory and hemodynamic statuses were not impaired.

**Figure 3:**
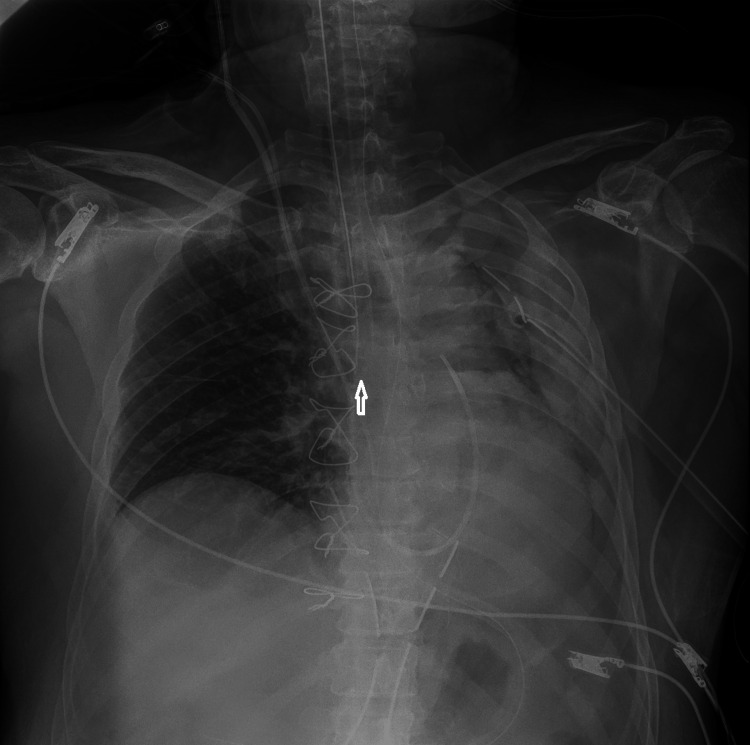
A left hemithorax drainage tube was applied; however, the pneumothorax was not resolved; instead, opacification of the left lung was observed. The tip of the endotracheal tube is positioned too low and is highlighted by an arrow.

**Figure 4:**
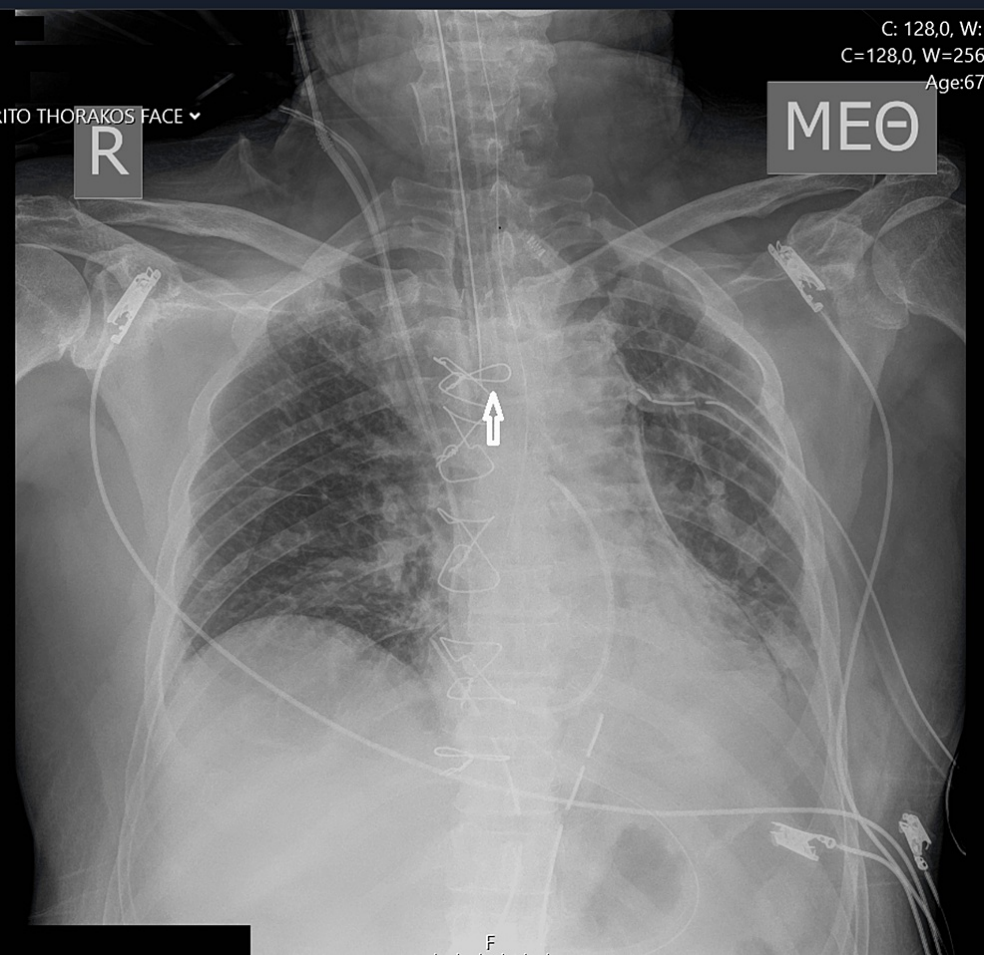
Endotracheal tube displacement is corrected. The arrow highlights the corrected position of the tube tip. The left lung is now completely expanded.

A 24-French chest tube was inserted through the 2nd intercostal space to drain the hemithorax. Shortly after, a second chest X-ray was ordered, revealing opacification of the entire left lung. The absence of breath sounds confirmed the chest X-ray findings. In the differential diagnosis, hemothorax and atelectasis were considered. Since the drainage tube was clear from blood and the patient's blood pressure was not impaired, the possibility of massive hemothorax was rejected. A closer examination of the X-ray revealed the endotracheal tube in a deeper position, likely inserted into the right main bronchus. Manipulations on the patient during the initial X-ray examinations could have caused the displacement of the endotracheal tube. Figure 3 displays the opacification of the left lung along with the unintended placement of the endotracheal tube into the right bronchus. Withdrawal of the endotracheal tube by three centimeters resulted in complete expansion of the left lung, as depicted in Figure 4.

## Discussion

Right mainstem intubation is relatively common due to the anatomy of the right main bronchus, which is wider, shorter, and more vertically oriented compared to the left main bronchus. This anatomical difference increases the likelihood of inadvertently inserting the endotracheal tube too far, leading to intubation of the right main bronchus. This misplacement can result in acute collapse of the left lung [4].

Unintentional endotracheal intubation may manifest as decreased expiratory flow and can lead to hypoxemia, difficult ventilation, atelectasis, contralateral pneumothorax, pneumomediastinum, and aspiration [3,5].

In our case, there was no left internal mammary artery (LIMA) preparation. The single bypass performed was a vein graft to the right coronary artery. Both pleurae were intact. Surgeons as well as the anesthesiologist were aware of side pleura movements after cardiopulmonary bypass disconnection; a pneumothorax did not exist until chest closure. Both lungs expanded completely after cardiopulmonary bypass disconnection.

The initial chest X-ray in the intensive care unit revealed a pneumothorax and a thorax drainage had to be applied. Following the thorax drainage, air bubbles immediately passed through the underwater seal in the chest drainage device (obviously the pneumothorax was drained) and there was no additional air leak thereafter.

No one could confirm the position of the endotracheal tube until the initial chest X-ray was reviewed (Figure 2). There was no sign of dysfunction in the ventilation machine or in the patient’s respiratory status.

When comparing Figure 2 to Figure 3, it becomes apparent that the endotracheal tube is positioned deeper in Figure 3, which is after the second radiogram. The patient was mobilized twice to have an X-ray. The endotracheal tube might be only a few millimeters displaced, even in Figure 2, but this cannot be documented. The most likely scenario might be that the tube was near displacement when the pneumothorax existed and it became fully displaced after the following second mobilization of the patient. A 1 or 2-mm withdrawal of the tube might be enough; however, we performed a 3-cm withdrawal to be sure the tube ends in the trachea.

The lung opacification after the left thorax drainage might be due to drainage tube kinking, hemothorax, obstruction due to mucus plug, or endotracheal tube mislocation. The use of ultrasound would have been decisively useful if the atelectasis had persisted.

It is not typically routine to perform a chest X-ray immediately after a patient's intubation in the operating room. Bilateral auscultation of the lungs is a commonly employed precaution that anesthesia providers should take to detect improper placement of the endotracheal tube. The tube must be adjusted according to the patient's dimensions and carefully secured at the proper distance at the incisors. Additional checks that are essential by the anesthetist to find out malplacement of an endotracheal tube include observation for symmetrical chest movements (rise and fall during positive pressure ventilation, indicating proper inflation of both lungs), colorimetric capnography (it measures the concentration of carbon dioxide in exhaled air), video laryngoscopy and fiberoptic bronchoscopy (direct visualization of the tube passing through the vocal cords), use of depth markers, and ultrasonography (used to visualize the trachea and confirm endotracheal tube placement, particularly in challenging cases or when other methods are inconclusive) [6-8]. Furthermore, the surgeon should consistently monitor the movements of the pleura on both sides, which are influenced by the inflation and deflation of the lungs following mid-sternotomy.

Early radiography should be a concern after the patient's admission to the intensive care unit following surgery [1].

## Conclusions

We present an unusual case of lung collapse in a patient intubated during heart surgery that persisted despite the insertion of a chest drainage tube. The cause was the simultaneous occurrence of pneumothorax and inadvertent intubation of the right mainstem bronchus, resulting in atelectasis of the left lung. It is likely that the endotracheal tube became displaced during the initial or the second radiograph in the intensive care unit. Subsequent X-rays promptly identified the position of the tube's tip and the atelectasis. Following partial withdrawal of the endotracheal tube, the chest radiograph returned to normal.

While dislocation of the endotracheal tube can occur and is well-documented in the literature, it is imperative that a patient undergoing heart surgery remains stable in both respiratory and hemodynamic statuses. Endotracheal tube dislocation may be due to malplacement from the beginning or displacement during patient transfer or mobilization for X-ray examination. The anesthesiologist should ensure the tube's position by securing its distance at the incisors and auscultating the lungs. Video laryngoscopy and fiberoptic bronchoscopy, use of depth markers, observation of chest wall, capnography, and ultrasonography can be particularly useful in difficult situations or when classical approaches yield uncertain results. As the endotracheal tube can become dislocated during patient transfer or mobilization for a radiograph, both the anesthesiologist and the intensivist should advocate for early radiography to detect pneumothorax and atelectasis. A complication can occur even in the most experienced medical staff; however, what is important is to be able to offer a solution. Employing multiple verification methods enhances patient safety. Double-checking significantly reduces the likelihood of errors, particularly in fields where accuracy and safety are paramount, such as health care.
